# Generation of rat offspring using spermatids produced through in vitro spermatogenesis

**DOI:** 10.1038/s41598-023-39304-1

**Published:** 2023-07-26

**Authors:** Takafumi Matsumura, Kumiko Katagiri, Tatsuma Yao, Yu Ishikawa-Yamauchi, Shino Nagata, Kiyoshi Hashimoto, Takuya Sato, Hiroshi Kimura, Takashi Shinohara, Makoto Sanbo, Masumi Hirabayashi, Takehiko Ogawa

**Affiliations:** 1 Department of Regenerative Medicine, Yokohama City University Graduate School of Medicine, Yokohama, Kanagawa 236-0004 Japan; 2 Research and Development Center, Fuso Pharmaceutical Industries, Ltd., 2-3-30 Morinomiya, Joto-ku, Osaka 536-8523 Japan; 3 Laboratory of Biopharmaceutical and Regenerative Sciences, Institute of Molecular Medicine and Life Science, Yokohama City University Association of Medical Science, Yokohama, Kanagawa 230-0045 Japan; 4 Department of Urology, Yokohama City University School of Medicine, Yokohama, Kanagawa 236-0004 Japan; 5 Micro/Nano Technology Center, Tokai University, Hiratsuka, Kanagawa 259-1292 Japan; 6 Department of Molecular Genetics, Graduate School of Medicine, Kyoto University, Kyoto, 606-8501 Japan; 7 Center for Genetic Analysis of Behavior, National Institute for Physiological Sciences, Okazaki, Aichi 444-8787 Japan

**Keywords:** Biological techniques, Cell biology, Developmental biology, Molecular biology, Physiology, Endocrinology

## Abstract

An in vitro spermatogenesis method using mouse testicular tissue to produce fertile sperm was established more than a decade ago. Although this culture method has generally not been effective in other animal species, we recently succeeded in improving the culture condition to induce spermatogenesis of rats up to the round spermatid stage. In the present study, we introduced acrosin-EGFP transgenic rats in order to clearly monitor the production of haploid cells during spermatogenesis in vitro. In addition, a metabolomic analysis of the culture media during cultivation revealed the metabolic dynamics of the testis tissue. By modifying the culture media based on these results, we were able to induce rat spermatogenesis repeatedly up to haploid cell production, including the formation of elongating spermatids, which was confirmed histologically and immunohistochemically. Finally, we performed a microinsemination experiment with in vitro produced spermatids, which resulted in the production of healthy and fertile offspring. This is the first demonstration of the in vitro production of functional haploid cells that yielded offspring in animals other than mice. These results are expected to provide a basis for the development of an in vitro spermatogenesis system applicable to many other mammals.

## Introduction

Spermatogenesis is a complex cell differentiation process supported by surrounding somatic cells, particularly Sertoli cells in the seminiferous tubules, along with cells outside the tubules, including peritubular myoid cells, Leydig cells, macrophages, and so forth. These testicular somatic cells, together with nutrients and hormones leaking from the vasculature as exudate, create a unique microenvironment for germ cells to undergo spermatogenesis^1^. The entire process takes 35, 50, and 74 days in mice, rats, and humans, respectively, and it can be divided into three phases: spermatogonial proliferation, spermatocytic meiosis, and differentiation of spermatids, i.e., haploid cells, into spermatozoa^2,3^. Recent advances in single-cell analysis technologies, such as single-cell RNA sequencing, have led to the accumulation of information on the molecular mechanisms of spermatogenesis^4^. However, in order to decipher the vital mechanisms of spermatogenesis and the pathology associated with its abnormalities, an experimental system that allows for close observation and intervention over a long period of time—namely, an in vitro experimental system—is required.

Research on mammalian in vitro spermatogenesis began a century ago^5^, and in 1937 it was reported that culturing the testis of newborn mice on a blood clot mixed with embryo extracts of fowl promoted spermatogenesis up to the meiotic pachytene stage^6^. More than 70 years later, in 2011, we succeeded in producing sperm from spermatogonial stem cells in cultured neonatal mouse testis tissues. Offspring were generated with those sperm and spermatids, demonstrating functional gametogenesis outside the body for the first time^7,8^. The culture medium used in the experiment was αMEM supplemented only with knockout serum replacement (KSR) (10% v/v). We then determined that a main ingredient in KSR—i.e., AlbuMAX, a bovine serum-derived albumin product—was responsible for the induction and maintenance of mouse spermatogenesis under our culture conditions^7^. This organ culture method was repeated by many researchers and is now established as the standard method for mouse in vitro spermatogenesis^9–13^.

Nonetheless, over the last decade it has become increasingly clear that this organ culture method is not effective in any animal species other than mice^5^. For example, when the testis tissues of cats^14^, marmosets^15,16^, and humans^17–20^ were cultured with this method, they failed to show spermatogenic progression. Indeed, in all these reports, the germ cells in the explants decreased as the culture progressed. As for rats, the historical work of the Steinbergers, who realized spermatogenesis in 4-days-old testicular tissue up to pachytene spermatocytes in 3 weeks, had long been the peak achievement^21,22^. Recently, Reda et al. reported spermatogenic progression up to the round spermatid stage (steps 5–6) by culturing 5-days-old pup testis tissue for 52 days with medium containing 10% KSR^23^. On the other hand, several other groups reported that rat in vitro spermatogenesis progressed no further than the pachytene spermatocyte stage^24–27^. Thus, in vitro spermatogenesis in rats remains a challenge.

Because AlbuMAX was the core contributor to the success of mouse in vitro spermatogenesis, we studied the components in AlbuMAX and found that lysophospholipids, triiodothyronine, testosterone, α-tocopherol, and retinoic acid were critical for promoting spermatogenesis^28^. When these reagents, along with ascorbic acid and glutathione as additional antioxidants, were added to a chemically defined medium, mouse spermatogenesis was induced even without KSR or AlbuMAX^28,29^. In addition to this work on optimization of the culture medium, we investigated the potential use of a microfluidic system for testis tissue culture^30,31^. We found that a silicone chip made of polydimethylsiloxane (PDMS) could be placed over the tissue to promote tissue culture, and we designated this approach the PDMS ceiling (PC) method^32,33^. Based on the results of these experiments, we cultured rat testis tissue and found that the modifications effective for rat testis tissue were the PC method, a lower concentration of oxygen and the addition of antioxidants and lysophospholipids. With these modifications, rat pup testis tissue cultured for 40 to 50 days successfully induced spermatogenesis up to the round spermatid stage^34^. However, the rat spermatogenesis in vitro did not reach the elongating spermatid stage, and the functionality of those round spermatids remained to be demonstrated.

In the present study, we introduced Acr-EGFP transgenic rats and cultured their testis tissue for the induction of spermatogenesis. Round spermatids production was monitored faithfully with GFP emission, and histological examination confirmed the elongating spermatids. Finally, the reproductive competence of the spermatids was confirmed by a microinsemination experiment.

## Results

### GFP fluorescence of Acr-EGFP transgenic rats

**Figure 1:**
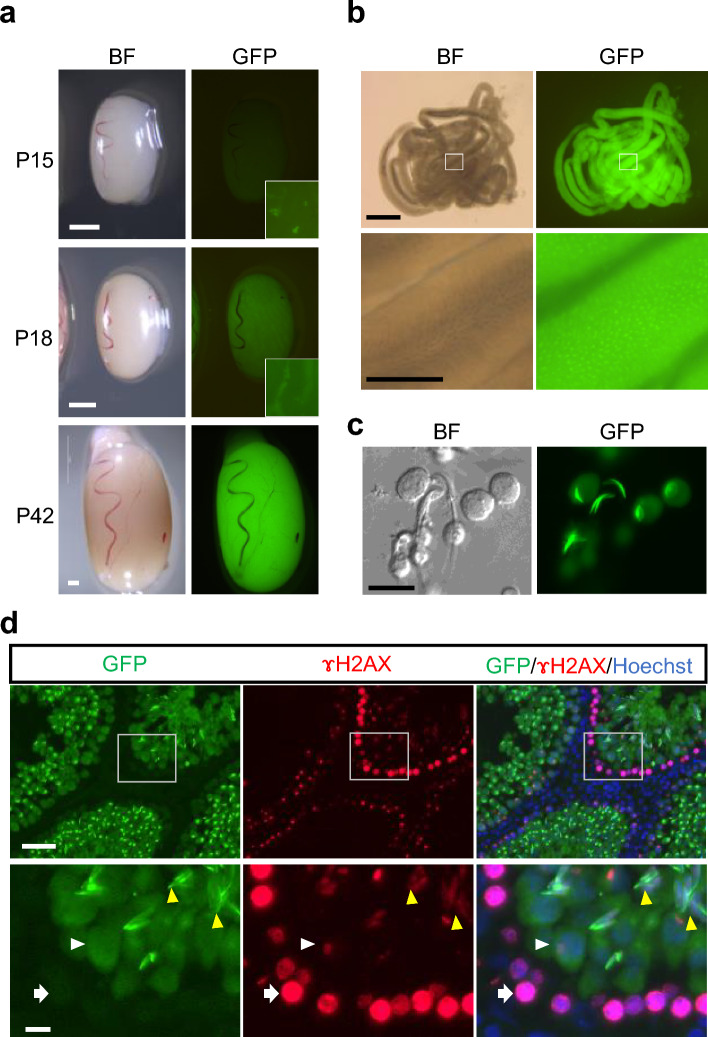
Characterization of the Acr-EGFP rat testis tissue. (**a**) The Acr-EGFP rat testis at postnatal days 15 (P15), P18, and P42 in brightfield (BF) and under GFP-excitation (GFP). GFP fluorescence was observed faintly on P15 and clearly on P18. Insets provide magnified views showing green fluorescence dot scattering on P15 and lining along the seminiferous tubules on P18. On P42, the GFP fluorescence grew stronger. (**b**) Seminiferous tubules of a 56 dpp rat showing strong GFP fluorescence. On closer observation with a stereomicroscope, the seminiferous tubules showed aligned strong GFP dots throughout the circumference of the tubule cylinder. (**c**) Released spermatids and spermatozoa showed GFP-positive acrosomes. (**d**) Immunohistochemistry of the testis tissue with antibodies to GFP and γH2AX along with Hoechst nuclear staining demonstrated that cells whose entire nuclei were yH2AX-positive, i.e., lepto-zygotene spermatocytes, were GFP-negative (white arrow), while the γH2AX-specked positive cells, i.e., pachytene spermatocytes, were GFP-positive (white arrowhead). In the center of the tubules, strong GFP aggregation in the acrosome was observed (yellow arrowheads). The rectangular areas in the upper panels are enlarged in the bottom panels. Scale bars: 2 mm (**a**), 1 mm (**b** *top*), 200 μm (**b** *bottom*), 20 μm (**c**), 50 μm (**d** *top*), 10 µm (**d** *bottom*).

We noticed in our previous experiments using Acr-GFP Tg mouse testes that acrosin-GFP was an excellent marker for identifying spermatids^35^. To take advantage of this marker, we introduced an *Acr*-EGFP transgenic strain of Sprague–Dawley (Slc:SD) rats^36^. The *Acr*-EGFP rat testis started to show EGFP fluorescence at around 2 weeks of age. On postnatal day 18 (P18), the testis showed weak GFP fluorescence under excitation light. On closer observation, cells emitting GFP fluorescence were observed in the center of seminiferous tubules. Later on, the fluorescence became stronger as the testis grew (Fig. 1a). When observing the dissociated seminiferous tubules of an adult *Acr*-EGFP Tg rat under excitation light, we found that the strong green fluorescence emitted from spermatids was aggregated into spots in a regularly arranged manner (Fig. 1b). When cells in the tubules were released, green acrosomes were recognized whose shape changed from a simple round spot to a crescent-like form along with spermatid maturation (Fig. 1c). Thus, this *Acr*-EGFP was confirmed to be an explicit and reliable marker of haploid rat cells. Next, we performed an immunohistochemical staining of the *Acr*-EGFP rat testis, using antibodies to GFP and γH2AX, which could differentiate cells in meiotic prophase into subclasses^37,38^. In these studies, GFP was not identified in leptotene-zygotene spermatocytes whose nuclei stained broadly with γH2AX, whereas it was identified in pachytene spermatocytes in which γH2AX was present as a small speckle in the nucleus (Fig. 1d). We also observed the aggregation of GFP in the acrosome in spermatids located in the inner region of seminiferous tubules (Fig. 1d). Together, these findings showed that *Acr*-EGFP was present in the cytoplasm of pachytene spermatocytes and condensed in the acrosome of spermatids, and thus *Acr*-EGFP is a useful marker for monitoring rat spermatogenesis.

### In vitro spermatogenesis using *Acr*-EGFP rat testis tissues

**Figure 2:**
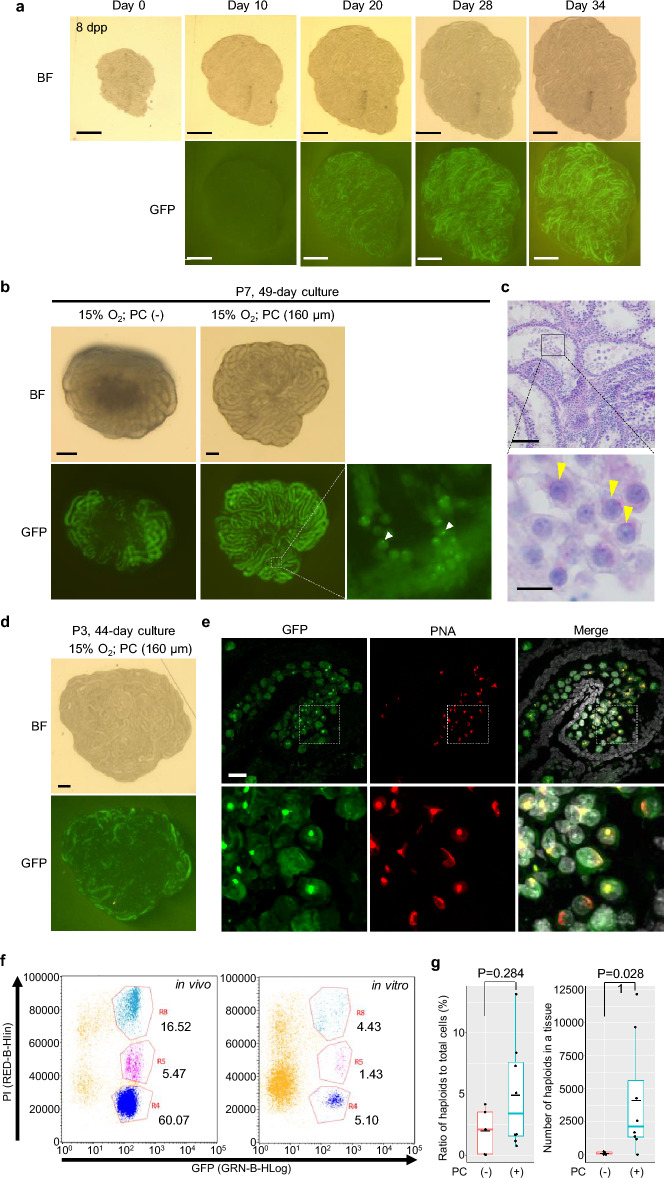
In vitro spermatogenesis in the cultured Acr-EGFP rat testis tissues. (**a**) Stereomicroscopic view of P8 rat testis tissues from culture day 0 to 34, using a PC chip with dent depth of 150 μm under 15% O_2_. Brightfield (BF) and GFP fluorescence (GFP) pictures were shown. (**b**) Stereomicroscopic view of P7 rat testis tissues cultured for 49 days, without and with a PC chip covering, left and center & right, respectively. The dent depth in the PC chip was 160 μm. Arrowheads indicate strong GFP aggregation in the acrosome in spermatids. (**c**) Histological section of a cultured tissue stained with PAS showed round spermatids having a PAS-stained acrosomal cap (yellow arrowheads). P7 rat testis tissue cultured for 43 days under a PC chip. The dashed rectangular area is enlarged in the bottom panel. (**d**) Stereomicroscopic view of P3 rat testis tissues cultured for 44 days; BF and GFP fluorescence pictures with a 160 µm-PC chip covering are shown. (**e**) Cryosection of the cultured tissue shown in (**d**) was stained with anti-GFP antibody (green), PNA for acrosome (red), and hoechst 33,342 for nuclear (white). The dashed rectangular area is enlarged in the bottom panel. (**f**) Flowcytometry of a dissociated cell suspension derived from testis tissue directly from an adult *Acr*-EGFP rat at 5 months of age (in vivo) and from the tissue of a P7 rat cultured for 50 days with a PC chip covering (in vitro). Numbers beside each area enclosed by a colored line represent the proportion of enclosed cells to total cells. R4: 1n; R5: 2n; R8: 4n. (**g**) Comparison of the haploid cell ratio in total cells and number of haploid cells per tissue between tissue samples treated with and those treated without a PC covering. Red and blue crossbars in the box indicate the median. Black crossbars indicate the average. P7–8 rat testis tissues were cultured for 49–55 days. The number of PC (−) tissues analyzed was 5 from 5 animals, and the number of PC (+) tissues analyzed was 8 from 4 animals. Scale bars: 1 mm (**a**), 500 μm (**b, d**), 50 μm (**c** *upper*), 10 μm (**c** *bottom*), 20 μm (**e**).

*Acr*-EGFP rats aged 3 to 9 days were used for testis tissue sampling. A testis was divided into 20 to 40 pieces of about 0.5–1.5 mm^3^ volume each. The pieces were cultured on an agarose gel block that was half-soaked in culture medium under 15% O_2_. The culture medium was designated rat medium 1, and its composition is shown in Supplementary Table S1^34^. When P7-8 rat testis tissues were cultured, seminiferous tubules emitting GFP fluorescence appeared at around 20 days and spread widely thereafter (Fig. 2a). The GFP expression was observed both with and without PDMS ceiling (PC) chip^34^ (Fig. 2b). However, the PC chip facilitated the horizontal expansion of the seminiferous tubules under the chip ceiling, which potentially enhanced the molecular exchange of nutrients, oxygen, and waste products. Under higher magnification, PC chip also proved advantageous in differentiating individual GFP-emitting cells. Some of these cells exhibited dense GFP aggregated into the acrosome, suggesting they were round spermatids (Fig. 2b). Regular histological examination with PAS staining demonstrated round spermatids in the center of the seminiferous tubules (Fig. 2c). Even when younger P3 rat testis tissues were used for cultivation, Acr-EGFP-positive cells appeared (Fig. 2d). Immunohistochemical staining of this sample with fluorescent conjugated lectin PNA and anti-GFP antibodies demonstrated round spermatids having a cap-shaped acrosome, indicating they were step 5–6 spermatids (Fig. 2e). In order to evaluate the spermatid production quantitatively, flow cytometry analysis was performed. In the testis of an adult rat (in vivo), about 60% of the cells were haploid cells. When a sample cultured with PC for 50 days was analyzed, haploid cells were determined to account for 5.1% of total cells (Fig. 2f). When comparing the effect of the PC chip, the ratio of haploid cells to total cells was higher in the PC group, but the difference was not significant. However, the actual number of haploid cells produced in a tissue was significantly higher in the PC group, demonstrating the advantage of the PC method (Fig. 2g).

### Sequential metabolomic analysis of culture media

**Figure 3:**
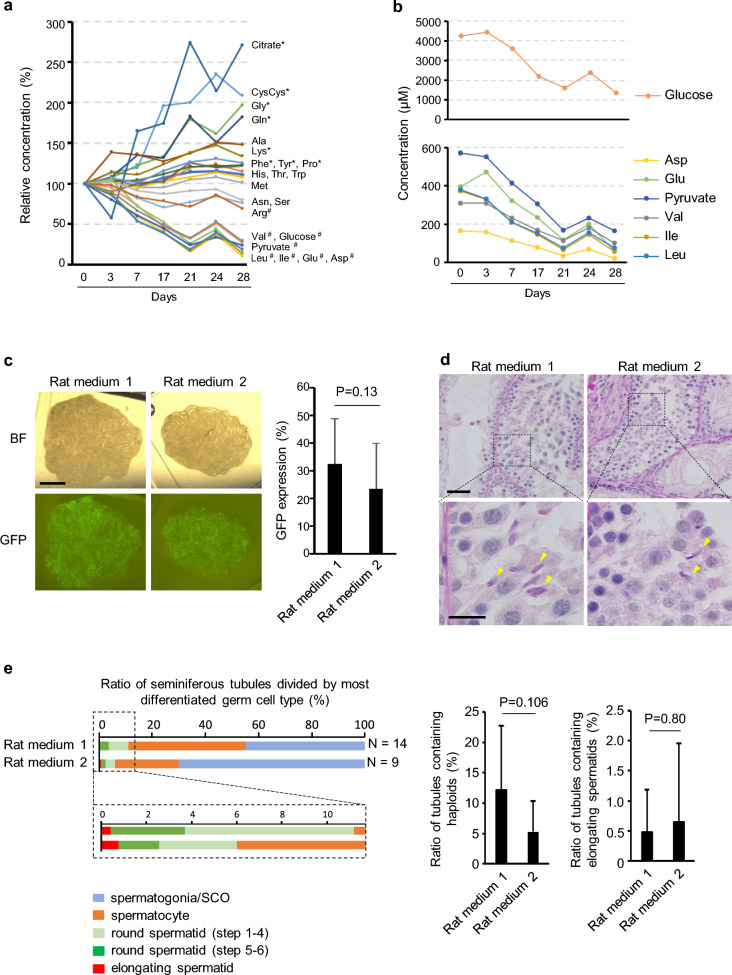
Metabolomic analysis of the culture medium and evaluation of new culture medium, Rat medium 2. (**a**) Summary graph showing changes in the concentrations of 23 substances, in the medium used for culturing P7 rat testis tissues, relative to those on day 0. Substances were classified into 3 groups according to a statistical analysis using Jonckheere-Terpstra test: substances showing increasing tendency (produced, marked by *), decreasing tendency (consumed, marked by ^#^), and the rest (stable). (**b**) Changes in the measured concentration of seven substances categorized into the consumed group are shown. P values by the Jonckheere-Terpstra test were 0.004 for Asp, Pyruvate and Ile, 0.01 for Glucose, Glue and Val and 0.02 for Leu. (**c**) P5 Acr-EGFP rat testis tissue cultured with either rat medium 1 or rat medium 2 for 31 days. Both showed broad GFP expression. Bar graph shows GFP expression area ratio cultured either with rat medium 1 or 2. Five rats aged P5 to P9 were used and the tissues were cultured for 36 to 45 days. Tissue number; n = 22 (rat medium 1) and 14 (rat medium 2). BF stands for brightfield. (**d**) PAS-stained histological view of P7 rat testis tissues cultured for 41 days. Yellow arrowheads indicate elongating spermatids. (**e**) Band graph showing the ratios of seminiferous tubules classified according to the most differentiated germ cell type contained. The round spermatids in steps 1–4 have dot-shaped acrosomes, while those of steps 5–6 have cap-shaped acrosomes. Bar graphs show average ratio and standard deviation of tubules containing haploids (left) or elongating spermatid (right) in total number of tubules observed. Six rats aged P5 to P9 were used and the tissues were cultured for 36 to 45 days. Tissue number; n = 14 (rat medium 1) and 9 (rat medium 2). Scale bars: 2 mm (**c**), 50 µm (**d** *top*), 10 µm (**d** *bottom*).

Next, we explored the metabolic dynamics of cultured testicular tissue by metabolomic analysis of components in the culture medium. P7 rat testis tissue was cultured, and the medium was collected on days 0, 3, 7, 17, 21, 24, and 28. On days 7, 21, and 28, the medium sample was collected before medium change. Among 50 substances measured, 23 substances were detected at concentrations over 1 μM, and these 23 substances showed different patterns of dynamic concentration change (Supplementary Tables S2–S3). The 23 substances were classified into 3 groups based on a statistical analysis using Jonckheere-Terpstra test: substances whose concentrations increased (produced), remained constant (stable), or decreased (consumed) as the culture proceeded (Fig. 3a, Supplementary Table S4). In our initial assessment of these findings, we simply conjectured that the consumed group would play an important role in the progression of spermatogenesis. In fact, it was noteworthy that the branched-chain amino acids of valine, leucin, and isoleucine were among the 6 consumed amino acids (Fig. 3b), because they have been reported to be selectively used as energy substrates by Sertoli cells^39^. We then supplemented the amount of each of these substances in rat medium 1 until their final concentrations were doubled compared to the initial formulation (Supplementary Table S5) and histologically examined their effect on increasing the formation of haploid cells. The ratio of seminiferous tubules containing round/elongating and elongating spermatids was measured (Supplementary Fig. 1a,b). Although none of the newly formulated media realized a significant increase in these ratios compared to rat medium 1, we decided to keep using the modified medium containing valine, leucine, isoleucine, glucose and ITS because of its potential advantage in promoting spermiogenesis (Supplementary Fig. 1b); we named this medium rat medium 2 (Supplementary Table S6). Rat medium 2 was as effective as rat medium 1 in inducing Acr-GFP expression in rat testis tissues, though it did not appear superior (Fig. 3c). Histological examination showed elongating spermatids both in tissues cultured with rat medium 1 and those cultured with rat medium 2 (Fig. 3d, Supplementary Fig. 2a). Then, we classified seminiferous tubules into 5 classes based on the most differentiated germ cells they contained: spermatogonia or Sertoli cells only (SCO), spermatocytes, round spermatids (steps 1–4), round spermatids (steps 5–6), and elongating spermatids (Supplementary Fig. 2b). Rat media 1 and 2 showed similar histological patterns, each supporting spermatogenesis up to the formation of elongating spermatids (Fig. 3e). Although these results did not establish the superiority of Rat Medium 2 over Rat Medium 1, we chose to continue using both. This decision was motivated by Rat Medium 2's potential advantage in facilitating the harvesting of haploid spermatids for microinsemination.

### Offspring production with microinsemination

**Figure 4:**
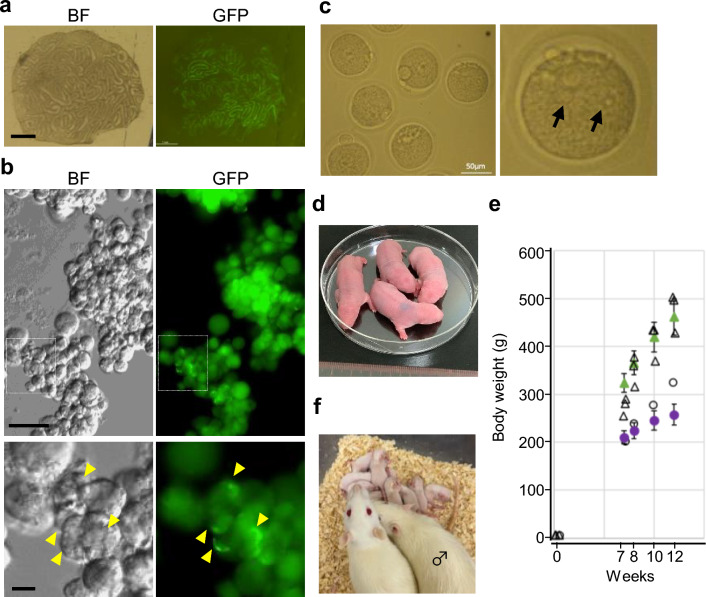
Offspring production with microinsemination. (**a**) A P8 rat testis tissue cultured under a PC chip with rat medium 2 for 47 days. BF stands for brightfield. (**b**) After dissociation, the testis tissue contained round spermatids showing GFP-positive acrosomes (arrowheads). The rectangular areas are enlarged in the bottom panel. (**c**) Oocytes fertilized with the round spermatids (ROSI), at 24 h after the procedure. On close observation, two pronuclei were identified (arrows). (**d**) Four pups were delivered by caesarean section. (**e**) Body weights of the 4 rats were monitored for 12 weeks, showing normal growth. Open circles and triangles indicate body weight of a female and 3 males of ROSI offspring. Green triangles and purple circles represent average body weight ± SD values of normal 20 male and 20 female SD (Crl:CD) rats, respectively, adapted from Kobayashi et al.^40^. (**f**) The next generation was produced by natural mating. A ROSI-derived male is indicated by a male (Mars) symbol. Scale bars: 1 mm (**a**), 10 μm (**b**), 50 μm (**c**).

After repeated observation of the spermatid production by in vitro spermatogenesis, we proceeded to the microinsemination experiments, using the round spermatid injection (ROSI) procedure. Testis tissues of P8 pups were cultured with either rat medium 1 or rat medium 2 for 47 days (Fig. 4a). The tissues were mechanically dissociated under a microscope using forceps. This procedure revealed numerous round spermatids exhibiting GFP fluorescent acrosomes in the samples cultured with rat medium 2 (Fig. 4b). Samples cultured with rat medium 1 also yielded spermatids, albeit in smaller quantities. The entire dissociated tissue sample, including the spermatids, was subsequently placed in a cryotubes containing CellBanker for cryopreservation. On the day of microinsemination, the cryopreserved sample cultured with Rat medium 2 was thawed, and round spermatids were collected under a fluorescent microscope. One hundred oocytes were fertilized with the spermatids and incubated for 24 h (Table 1). Seventy-six were viable the next day, with 2 pronuclei observed in 67 oocytes and cleavage observed in 6 (Fig. 4c). All 76 fertilized eggs were divided and transferred to 3 pseudo-pregnant females. On 21 days after the transfer, caesarean section revealed 14 implantation marks and 4 live offspring (3 males and 1 female) (Fig. 4d). One male pup had the EGFP transgene, demonstrating the transfer of the hemizygous EGFP gene from the original Tg rat (Table 1). All 4 pup rats grew healthily and showed a standard increase in body weight (Fig. 4e). After maturation, they were mated for breeding; 2 males showed fertility, and their mates delivered the next generation (Fig. 4f, Supplementary Table S7).

**Table 1:** Summary of the microinsemination experiment.

No. (%) of oocytes						No. (%) of pups	
injected	Survived	Foemed 2PN	Cleaved	Transferred	Implanted	Born	With EGFP
100	76 (76)	67 (88)	6 (8)	76	14 (18)	4 (5)	1 (25)

One hundred oocytes were each injected with a spermatid obtained from the cultured testis tissues. Of these, seventy-six oocytes survived and 67 displayed 2 pronuclei (PN). Cleavage was observed in 6 of these oocytes. The 76 fertilized eggs were then transferred to 3 pseudo-pregnant females. Caesarean section revealed 14 implantation sites and resulted in the birth of 4 live offspring (3 males and 1 female). One of the male pups carried the EGFP transgene.

## Discussion

In this study, we have shown that rat haploid cells produced under our culture conditions were able to produce healthy and fertile offspring. This is the first demonstration of the fertility and safety of male gametes produced in vitro in animals other than mice.

In our previous study, we developed a method for rat in vitro spermatogenesis through several modifications on medium and other culture conditions that were originally developed for mice^34^. That experiment was performed with a line of transgenic rats, Haspin-Venus (HV-Tg), whose strain background was Wistar. The modifications of the culture conditions were efficiently evaluated by monitoring the Venus expression in the cytoplasm of meiotic germ cells. In the present study, the culture method was tested and confirmed to be effective in a different line of transgenic rats, Acr-EGFP-Tg, with a different strain background, Sprague–Dawley. The EGFP fluorescence in the acrosome facilitated the identification and collection of round spermatids, leading to successful production of offspring by ROSI. The Acr-EGFP was also useful for the identification of elongating spermatids. To our best knowledge, elongating spermatid production in vitro in rats is also a novel achievement, along with recent publication by another research group^41^.

It is well known that the cells of rats are more difficult to handle and manipulate under culture conditions than those of mice, although the reasons for the difficulty are unclear. For instance, rat ES cells were established in 2008, about 30 years after the corresponding line in mice^42,43^. As for intracytoplasmic sperm injection (ICSI), normal live offspring were first obtained in rabbits in 1988^44^, followed by cattle in 1990^45^, humans in 1992^46^, mice in 1995^47^, sheep in 1996^48^, pigs in 2000^49^, and finally rats in 2002^50^. Nonetheless, through the dedicated efforts of various research groups, along with advances in culture techniques and other technologies, some progress has been made in rat cells. As a result, it was recently reported that primordial germ cell-like cells were generated in vitro from pluripotent stem cells (PSCs) of rats, albeit a decade later than in mice^36^. This achievement, together with that of our present study, is encouraging for the future development of a total in vitro differentiation system from PSCs to sperm, as shown in a mouse system^51^.

In this study, we also tried to decipher the metabolic dynamics in the cultured testis tissue by measuring concentration changes in the major components of the culture medium during a tissue culture experiment. To our surprise, many of the 50 substances measured showed dramatic concentration changes during the 28 days cultivation. We speculated that the 7 substances that were significantly decreased in concentration over time—glucose, pyruvate, aspartic acid, glutamic acid, valine, leucine, and isoleucine—would be actively consumed by the testis tissue, suggesting that they play important roles in spermatogenesis. In particular, among all amino acids, valine, leucine, and isoleucine were drastically decreased during the cultivation. This finding would be in agreement with a report that those three branched-chain amino acids were taken up selectively by cultured primary rat Sertoli cells and served as their main energy source^1,39^. Therefore, the present metabolomic data seemed to well reflect the metabolic dynamics of testis tissue. Although we observed no clear difference in spermatogenesis when these consumed substances were simply added to the medium, it is yet useful studying the metabolism of testis tissue with the use of metabolomics of the medium, which could potentially assist in the further optimization of the culture medium for in vitro spermatogenesis. If different animal species have different metabolic characteristics, metabolomic analysis should help optimize the culture medium for each species.

We demonstrated that rat spermatogenesis progressed up to the elongating spermatid stage under our culture condition. In addition, using round spermatids produced in vitro, healthy and reproductively competent offspring were generated through microinsemination. Although we experienced significant species differences in spermatogenesis between mice and rats, the culture techniques used in the present rat experiments should be applicable to other animals as well, which could lead to the development of a general method of in vitro spermatogenesis.

## Methods

### Animals

Acrosin-EGFP transgenic rats (Sprague–Dawley strain) were produced^36^ and provided by M.H. The genotype was determined by PCR using the following primer set detecting the *gfp* gene and KOD DNA polymerase (KOD One; KMM-101; TOYOBO, Osaka, Japan). Forward primer: 5′-AAGGGCATCGACTTCAAGG-3′. Reverse primer: 5′-TGCTTGTCGGCCATGATATAG-3′. Wild-type Slc:SD rats were purchased from Japan SLC (Shizuoka, Japan). *Haspin*-Venus transgenic (HV-Tg) rats (Wistar strain) were maintained in our animal facility^34^. Rats were housed in air-conditioned rooms at 24 ± 1 °C and 55 ± 5% humidity, under a 14 h/10 h light/dark cycle. Animals were kept in a specific pathogen-free room. MF hard pellets (Oriental Yeast, Tokyo) were fed ad libitum. Drinking water was acidified to pH 2.8–3.0 by HCl. All animal experiments were performed in accordance with the Guidelines for Proper Conduct of Animal Experiments (Science Council of Japan) and were approved by the Institutional Committee of Laboratory Animal Experimentation (Animal Research Center of Yokohama City University; protocol nos. F-A-20-038). The study is reported in accordance with ARRIVE guidelines.

### Culture method

The culture method has been described previously^7,34,35^. Briefly, the testes of rats, postnatal days 3–9 (P3–P9), were decapsulated and gently separated by forceps into 20 to 40 pieces per testis. The tissue fragments were then placed on a block of 1.5% agarose gel half-soaked in the well of a 12 well-culture plate containing 0.5 mL of culture medium. Before culture, the gel block was submerged twice in medium, for more than 6 h each time, with a medium change in between. Each tissue piece on the agarose gel block was 1–3 mm in diameter and around 200 µm in thickness. Each gel block was loaded with 3 to 4 tissues. In cases in which the PC method was applied, a single tissue was placed on a gel, and a PC chip with an indentation depth of 160 µm was used to cover the tissue. The PC was prepared as described in previous papers^32,52^. The medium was changed once a week. The culture incubator was supplied with 5% carbon dioxide and 15% O_2_ and maintained at 34 °C. Cultured tissues were observed once a week under a stereomicroscope equipped with an excitation light for GFP (LeicaM205 FA; Leica, Wetzlar, Germany). The extent of GFP expression in each tissue sample was assessed on a scale of 0–100% (0, 5, 10, 20, 30, 40, 50, 60, 70, 80, 90, and 100%), judging visually the ratio of the area of GFP expression in the whole region of each tissue fragment.

### Culture medium preparation

Rat medium 1 was prepared as described previously with some modifications^34^. Briefly, AlbuMAX I (11020-021; Thermo Fisher Scientific, Waltham, MA, USA) was dissolved in double-concentrated α-MEM (12000-022; Thermo Fisher Scientific) at half the volume of the final solution, stirred until completely dissolved, and then supplemented with the following factors at the concentrations indicated in Supplementary Table S1: testosterone (20808341, Wako; 10 mM stock solution in ethanol), triiodothyronine (T6397, Merck; 2.0 µg/mL stock solution in ethanol), LH (L5259, Merck; 50 µg/mL stock solution in Milli-Q water), FSH (F4021; Merck, 50 µg/mL stock solution in Milli-Q water), L-ascorbic acid 2-glucoside (092375; Matrix Scientific, 1 M stock solution in Milli-Q water), α-tocopherol acetate (47786, Merck; 1 M stock solution in ethanol), L-glutathione (G6013, Merck; 100 mM stock solution in Milli-Q water), L-a-lysophosphatidylcholine (LPC) (L4129, Merck; 50 mg/mL stock solution in ethanol), and lysophosphatidic acid (LPA) (L7260, Merck; 10 mM stock solution in 10% BSA). Then, a 7% NaHCO3 solution was added to achieve a final concentration of 1.82 g/L. Antibiotic–antimycotic (15240062; Thermo Fisher Scientific) was added at a 1/100 volume to achieve a final concentration of 100 IU/ml for penicillin, 100 µg/mL for streptomycin, and 250 ng/ml for amphotericin. For rat medium 2, D(+)-glucose (049-31165; Wako, Osaka, Japan), ITS-G (41400-045; Thermo Fisher Scientific), L-valine (V0500-25G; Merck, Darmstadt, Germany), L-isoleucine (I2752-10G; Merck), and L-leucine (L8000-25G; Merck) were added to rat medium 1 at the concentrations indicated in Supplementary Table S3. After sterilization with a Millipore filter having a pore size of 0.22 µm, the media were stored at 4 °C.

### Histological and immunohistochemical examinations

In histological examinations, specimens were fixed with Bouin’s fixative and embedded in paraffin. Sections for each specimen were stained with hematoxylin and periodic acid Schiff (PAS). The stage of germ cell development was identified by the morphology and the staining pattern of the acrosome and nucleus^3^. In immunofluorescent staining, tissues were fixed with 4% paraformaldehyde (PFA) in PBS at 4 °C overnight. Tissues were then soaked in successive solutions of 10%, 15%, and 20% (w/v) sucrose in PBS for 1 h each for cryoprotection. They were then cryo-embedded in OCT compound (Sakura Finetek Japan, Tokyo) and cut into 7 µm thick sections. The cryosections were washed with PBS and treated with methanol for 10 min, and then four times with 0.2% PBT (0.2% Triton X–100 in PBS) for 10 min each time, and finally with Image-iT FX Signal Enhancer (Thermo Fisher Scientific) for 30 min. Incubation with primary antibodies diluted in Can Get Signal immunostain Solution A (TOYOBO, Osaka, Japan) was performed overnight at 4 °C, followed by rinsing four times with PBS, and then secondary antibodies diluted in Can Get Signal immunostain Solution A were applied for 1 h at room temperature. The sections were washed with PBS and nuclei were counterstained with Hoechst 33,342 dye. Finally, the sections were mounted using Prolong Diamond Antifade Mountant (Thermo Fisher Scientific) before observation. The antibodies used as primary antibodies were anti-GFP (1:3000; ab13970; Abcam, Cambridge, UK) and anti-ɤH2AX (1:1000; ab81299; Abcam). The antibodies used as secondary antibodies were Alexa Fluor 488-conjugated goat anti-chicken antibody (1:200; A–11039; Thermo Fischer Scientific) and Alexa Fluor 555-conjugated goat anti-rabbit antibody (1:200; A–21428; Thermo Fischer Scientific). Lectin PNA From Arachis hypogaea (peanut), Alexa Fluor™ 568 Conjugate (1:1000, L32458; Thermo Fischer Scientific) was used to identify acrosome.

### Flow cytometric analysis

The flow cytometric analysis was conducted according to previous reports^32,53^. Testicular tissues were digested with 1 mg/mL collagenase, 0.25% trypsin, and 1 mg/mL DNase I in PBS for 25 min at 37 °C with additional mechanical agitation every 5–10 min by pipetting or tapping. After stopping the reaction by adding Dulbecco’s modified Eagle’s medium containing 10% FBS, the cells were filtered using a 40 μm pore size cell strainer (Becton Dickinson, Franklin Lakes, NJ), pelleted by centrifugation (220* g*, 5 min) and resuspended in PBS containing 3% (vol/vol) FBS. The cells were fixed by 2% PFA in PBS for 20 min on ice and washed twice by PBS. Then the cells were treated with 70% ethanol and incubated at 4 °C overnight. After centrifugation (2200*g*, 5 min), the cell pellet was washed twice with 0.2% PBST [PBS containing 0.2% Triton X-100] containing 10% FBS. Next, the cells were stained by Alexa Fluor 488-conjugated GFP polyclonal antibody (A-21311; Thermo Fisher Scientific) diluted with 0.2% PBST containing 5% BSA (1:300) at 37 °C for 1 h. The cells were washed with 0.2% PBST + 10% FBS twice. Then, the supernatant was removed and stained with 300 µL PI staining solution [20 µg/mL PI stock (P4864-10ML; Merck), 200 µg/mL RNase A (Nacalai Tesque, Kyoto, Japan) in 0.2% PBST] at 37 °C for 15 min.

The samples were applied to a Guava easyCyte flow cytometer (Merck, Darmstadt, Germany). First, viable single cells were identified and selected by propidium iodide and profiling by forward and side scatter. Then, they were separated by GFP expression. The number of haploids was calculated using the ratio of haploids to the total cell concentration and volume of samples measured by easyCyte. Data plotting was performed using Rstudio ver. 1.4.1717.

### Metabolomic analysis

Four testicular fragments from a P7 HV-Tg rat were placed on a 1.5% agarose gel block without a PC device as described above. The first day of culture was designated as day 0. 30 µL of medium was collected from each well on days 3, 7, 14, 17, 21, 24 and 28. On days 7, 14, 21 and 28, the culture medium was fully changed after collecting the sample. The LC/MS/MS method package for cell culture profiling (version 2; Shimadzu, Kyoto, Japan) was used for the metabolomics analysis. Briefly, 40 µL of Milli-Q water, 10 µL of 0.5 mM isopropylmalic acid (333115, Sigma) as an internal standard, and 100 µL of acetonitrile (018-19853, Fujifilm, Tokyo) were added to 10 µL of standard or sample medium and vortexed. 50 μL of the supernatant of the sample after centrifugation at 15,000 rpm for 15 min at room temperature was collected and diluted with 450 μL of Milli-Q water, and 1 μL was introduced into an LC (Nexera X3; Shimadzu) with a Discovery HS F5-3 column (567503-U, Merck; 2.1 mm I.D. × 150 mm, 3 μm) connected to a tandem quadrupole mass spectrometer (LCMS-8060NX; Shimadzu). The chromatographic parameters were set according to the instruction manual (225-41626A; Shimadzu). LabSolutions Insight (Version 3.7 SP3; Shimadzu) was used to analyze the chromatographic data.

### Microinsemination

Cultured rat testis pieces showing EGFP fluorescence were dissected by fine forceps. The fragmented tissues were put in a serum tube containing 1 ml of Cellbanker 1 solution (Nippon Zenyaku Kogyo, Japan). The tubes were then placed in a deep freezer until use. On the day of microinsemination, the cryopreserved samples were thawed and placed in a dish to find and collect the round spermatids having a GFP-positive acrosome. Round spermatid injection (ROSI) was carried out with a Piezo-driven micromanipulator, as previously described^54^. Oocytes retrieved from superovulated Slc:SD female rats at 4–5 weeks of age were activated with 5 µM ionomycin in mR1ECM for 5 min followed by an additional 40 min of culture in mR1ECM medium^55^ before ROSI, and then the ROSI oocytes were treated with 5 μg/ml cycloheximide in mR1ECM for 4 h. The next morning, the ROSI oocytes were transferred into the oviducts of pseudopregnant Crlj:WI (Charles River Japan Inc., Kanagawa, Japan) recipients. Offspring were recovered by cesarean section at 21 days after oocytes transfer.

### Statistical analysis

A non-parametric comparison test, using the Mann–Whitney U method, was conducted using R software (ver. 4.0.3) to assess the significance of differences in the ratio and number of haploids produced in vitro (Fig. 2g). The Jonckheere-Terpstra test was applied to evaluate trends in the metabolome data during the culture period (Fig. 3a,b, Supplementary Tables S3, S4). The Student’s t-test was conducted to assess the significance of differences in the GFP expression area ratio (Fig. 3c). The Wilcoxon rank sum test was employed to test the ratio of seminiferous tubules containing haploids or elongating spermatids within the cultured tissues (Fig. 3e). Lastly, the Kruskal–Wallis test was performed to assess the significance of differences in the ratio of seminiferous tubules containing haploids or elongating spermatids within the cultured tissues (Supplementary Fig. 1a,b).

## Supplementary Information

Supplementary Information.

## Data Availability

The datasets used and analyzed during the current study are available from the corresponding author upon reasonable request.
