# Supplementary material for: Generation of rat offspring using spermatids produced through in vitro spermatogenesis
Source: Sci Rep. 2023 Jul 26;13:12105. doi: 10.1038/s41598-023-39304-1 (PMC10372019; doi:10.1038/s41598-023-39304-1)
Supplement: Supplementary file 1 — Supplementary Information. [file 41598_2023_39304_MOESM1_ESM.pdf]

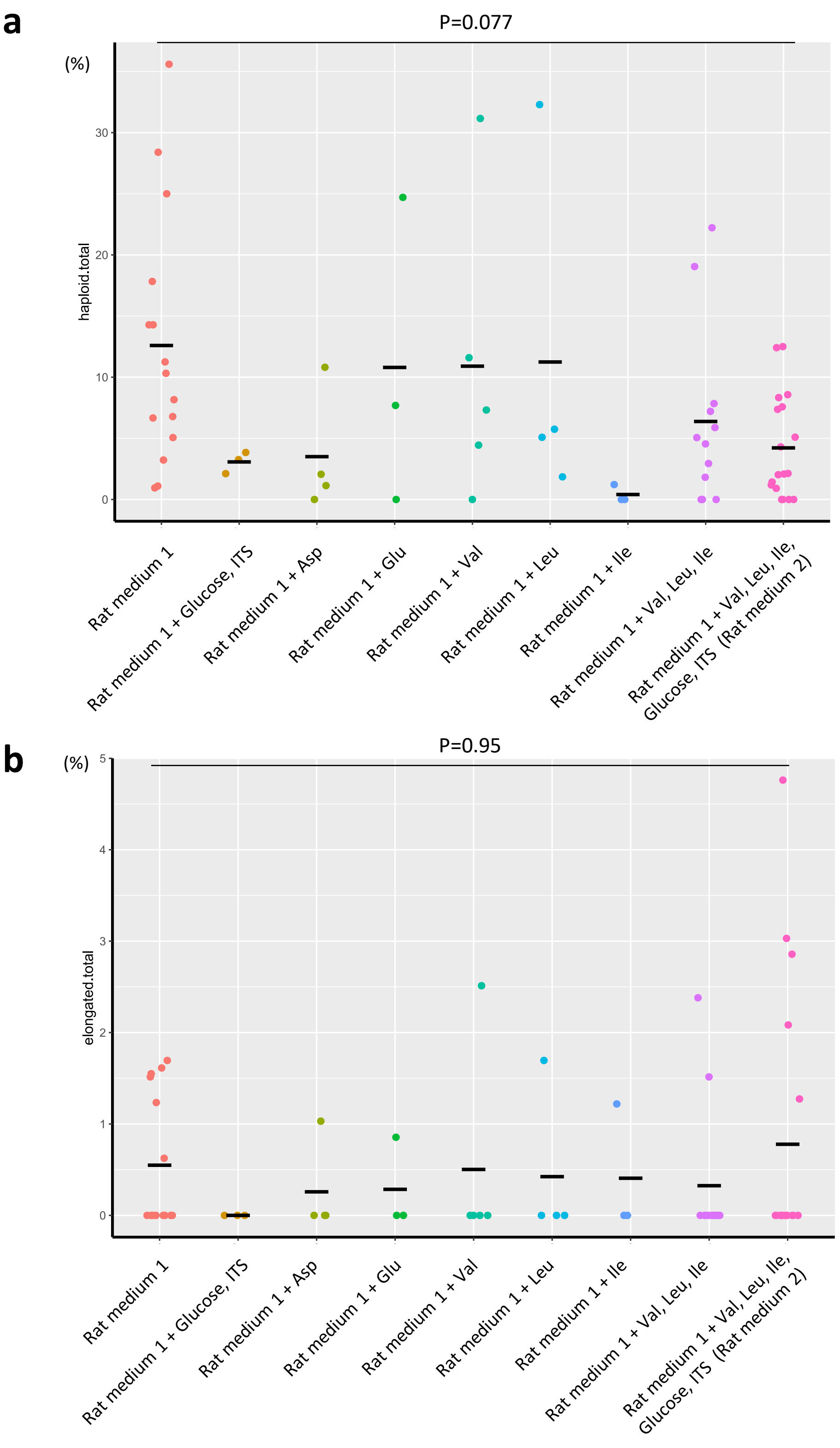

Supplementary Figure 1: Augmentation of consumed substances in rat medium 1.

(**a**) Rat medium 1 was supplemented with substances as indicated in bottom column and used for culture experiments. Cultured tissues were histologically examined, and seminiferous tubules containing round or elongating spermatids were counted as positive. The positivity rates were calculated by dividing the number of positive tubules by the number of total tubules examined. Crossbars indicate mean values. Number of animals used in the experiment was seven. (**b**) The rate of elongating spermatid-containing seminiferous tubules was calculated in the same manner as in (**a**). ITS: insulin-transferrin-selenate; Asp: aspartate; Glu: glutamic acid; Val: valine; Leu: leucine; Ile: isoleucine. Statistical significance is shown as P value on the top of each graph.

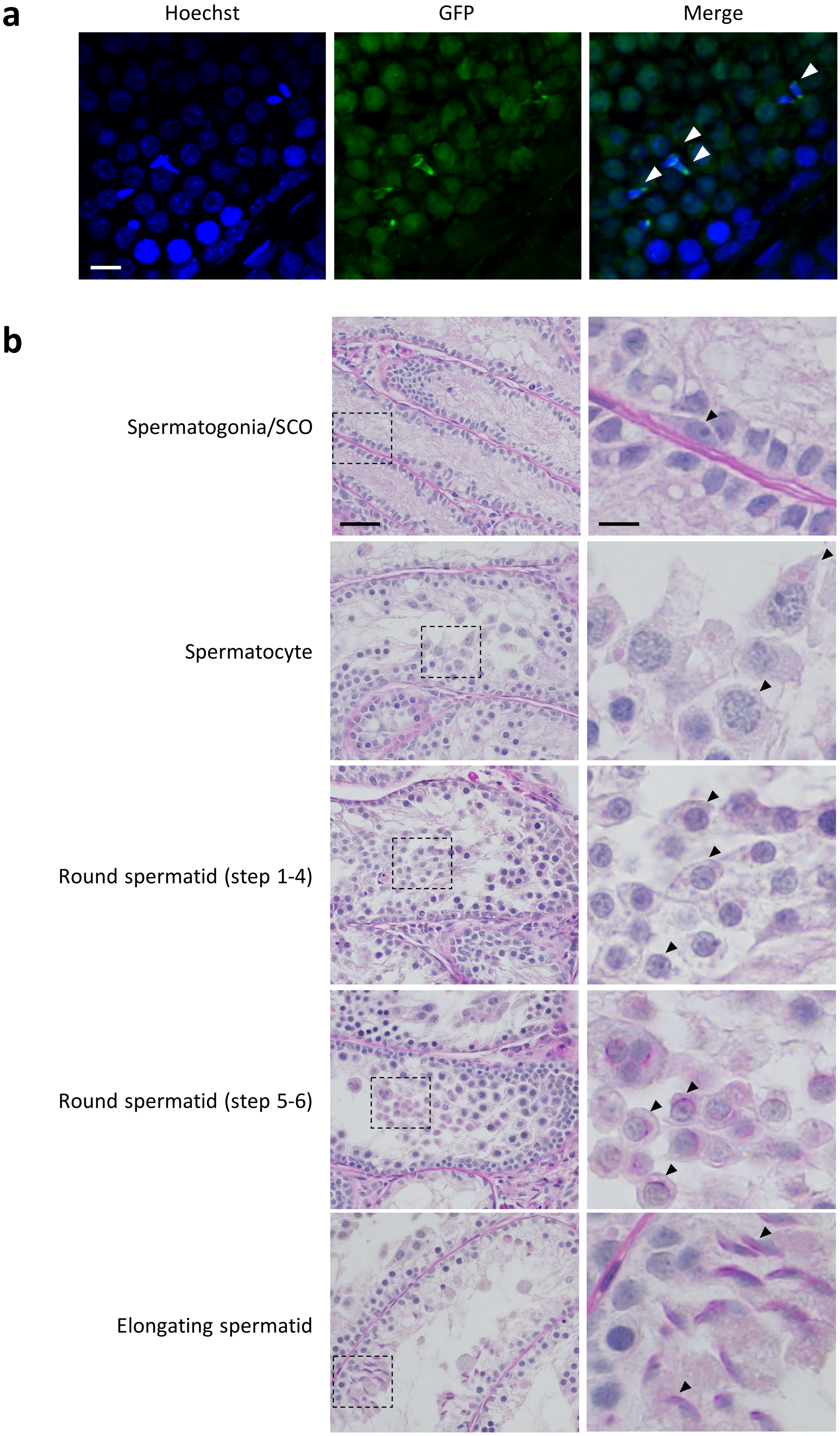

Supplementary Figure 2: Histological analysis of cultured tissues.
(**a**) A P7 rat testis tissue was cultured under a PC chip with rat medium 1 for 57 days. Immunostaining with anti-GFP antibody (green) and Hoechst 33342 nuclear staining (blue). (**b**) PAS-stained view of germ cells in P7 rat tissues cultured for 41 days using rat medium 1. Scale bars: 20 µm (*A*), 50 µm (*B left*), 10 µm (*B right*). Arrowheads indicate the cell type written to the left of the panel [spermatogonia, spermatocyte, round spermatid (step 1-4), round spermatid (step 5-6), elongating spermatid], respectively.

Rat medium 1		
AlbuMAX I	20	mg/mL
Testosterone	1	µM
3,3′,5-Triiodo-L-thyronine sodium	2	ng/mL
LH	1	ng/mL
FSH	1	ng/mL
L-Ascorbic acid 2 glucoside	0.5	mM
DL-α-Tocopherol acetate	0.5	mM
L-Glutathione reduced	0.5	mM
L-α-Lysophosphatidylcholine	100	µg/mL
Lysophosphatidic acid	10	µM

Ingredients were added to the α-MEM.

Supplementary Table 1: Composition of rat medium 1

Components	Day 0	Day 3_wo	day 7_wo	Day 3	Day 7	Day 17	Day 21	Day 24	Day 28
Thr	342	361.9	371.5	378.7	359.7	388.6	427.6	419.4	415.1
Cys	760.3	571.6	439.3	579.9	460	244.7	511.3	528.5	543.2
Ala	203.7	220.9	226.4	307.3	308.4	282.6	311.7	332.9	337
His	140	158.2	166.5	167	169.7	160.9	199.7	196.9	192.2
Met	71.2	76.5	78.9	79.1	75.5	73	83.5	83.2	80.4
Tyr	139.8	143.7	139.8	149	146.1	161.2	169.9	176	172.3
Cyanocobalamin	0.9	0.9	0.9	0.9	0.9	0.9	1.1	1	1
Folic_acid	1	1.1	1.1	1	1	1	1.1	1.1	1.1
Trp	33.8	33.4	34	34.8	32.3	34.5	36.8	38.2	36.8
Threonic_acid	7.9	25.7	27.9	26.8	30	33.7	43.7	38.8	43
CysCys	48.8	68.5	86.7	78.2	103.9	134.4	173.4	161.3	180.9
HyPro		0.5	0.4	1.6	3.5	3.7	5.8	4.3	4.9
Gly	427.6	523.7	507.6	564.3	625.6	701.9	908.9	846.6	999.7
Gln	1282.1	1228.8	1055.1	1334.5	1438.2	1625.4	1926.1	1857	1922.2
Methionine_sulfoxide	1.4	2.7	3.3	2.5	3.4	3.5	4.3	4.7	4.6
Cit		0.4	0.5	2.5	4	3.4	4.3	3.4	3.5
Orn	0.2	0.7	0.7	7.9	15.1	9.8	10.1	8.9	9.8
Pro	238	280.7	259.5	253.8	260.9	289.2	328.1	338.7	317.6
2AE				12.8	14	15	25.3	22.7	23
Lys	262.1	264.2	286.5	302	318.7	328.2	397.5	390.8	385.1
Lactate				435.5	967.9	1050.8	1254.5	1155.4	1091
Uracil				1.4	6.8	10.7	15.5	9.2	10.1
Citrate	16.7	20.7	19.9	11.9	32.9	36.1	54.7	44.4	54.1
Uric_acid				1.5	13	18.5	32.9	19.4	28
Opro	93	263.6	458	230.3	402.7	448.8	733.7	609.5	773.6
Uridine		0.7	0.7	2.5	2.8	4.4	6	4.8	5.7
Choline	5.9	8.2	11.8	17.9	22.2	17.4	24.9	20.6	24
Putrescine	0	0	0.8	6.2	19.3	14.1	19.3	11.7	12.3
Thymidine				2.5	3.9	4.9	6.6	4.8	6.6
Cytidine		0.7	0.8	2.9	4.3	4.1	5.7	4.3	5.2
Deoxycytidine		0.1	0.1	2.7	4.8	4.6	6.1	4.4	5.2
Biotin	0.5	0.4	0.4	0.4	0.5	0.4	0.4	0.4	0.6
Phe	133.1	132.9	137.6	143	140.1	154.2	174.3	174.3	172.9
Glucose	4253.9	4669.5	4961.3	4450.3	3620.6	2196.7	1598.8	2386.2	1361.5
Asn	571.8	558.6	561.7	574.6	515.9	491.2	512.2	521.9	448
Asp	166.6	187.1	194.3	159.6	113.4	77.4	32.2	69.2	19.7
Ser	158.2	181.3	182.7	178	150.4	128.9	141.7	153.9	139.3
Glyceric_acid		17.9	21.7	16.7	15.1	13.7	18.5	18.7	12.5
Glu	397.2	448.7	448.1	472.9	321.6	236.2	116.2	199.2	59.4
Ascorbate	81.1	0	0	3.6	0	0	0	0	0
Pyruvate	571.4	688.1	686.9	552.6	414.7	307	168.4	231.6	165.9
Arg	425.2	437.2	465.3	428.9	389.7	344.9	335.2	378.2	324.1
Glutathione	40.9	32.2	26.7	32.3	22.7	19	20.3	21	14.5
Val	307.8	340.6	350	308.2	232.5	166.9	114.6	181.9	102.4
Niacinamide	6.3	6.5	6.7	6.6	5.6	5.2	5.1	5.5	4.2
Oxidized_glutathione	486	463.3	439.2	461.1	418.4	401.3	375.9	391	316.7
Riboflavin	0.5	0.4	0.5	0.4	0.4	0.4	0.5	0.3	0.3
Pyridoxal	8.1	6.1	4.2	6.4	4.3	3.3	2	2.6	1.4
Ile	373.7	369.4	393.8	327.2	211.5	146.2	65.6	144.6	57.4
Leu	379.4	381.9	393.6	332	211.2	150.8	74	154.2	75.2

Supplementary Table 2: Concentrations of 50 medium components (µM). The data presented in the shaded columns of Day 3_wo and Day 7_wo represent the concentrations of medium components in wells without tissue. These serve as baseline controls for Day 3, 17, 24 and Day 7, 21, 28, respectively. The values for Day 3, 17, and 21 were adjusted by multiplying them with the ratio (Day 0/Day 3_wo), while the values for Day 7, 21, and 28 were adjusted by multiplying them with the ratio (Day 0/Day 7_wo). The adjusted values are provided in Supplementary Table 3.

Components	Day 0	Day 3	Day 7	Day 17	Day 21	Day 24	Day 28	P value
Thr	342	357.9	331.1	367.2	393.6	396.3	382.1	0.05
Cys	760.3	771.3	796.1	325.5	884.9	703.0	940.1	0.29
Ala	203.7	283.4	277.5	260.6	280.4	307.0	303.2	0.1
His	140	147.8	142.7	142.4	167.9	174.2	161.6	0.1
Met	71.2	73.6	68.1	67.9	75.4	77.4	72.6	0.45
Tyr	139.8	145.0	146.1	156.8	169.9	171.2	172.3	0.002
Cyanocobalamin	0.9	0.9	0.9	0.9	1.1	1.0	1.0	0.09
Folic_acid	1	0.9	0.9	0.9	1.0	1.0	1.0	
Trp	33.8	35.2	32.1	34.9	36.6	38.7	36.6	0.07
Threonic_acid	7.9	8.2	8.5	10.4	12.4	11.9	12.2	0.01
CysCys	48.8	55.7	58.5	95.7	97.6	114.9	101.8	0.004
HyPro		0.0	0.0	0.0	0.0	0.0	0.0	
Gly	427.6	460.7	527.0	573.1	765.7	691.2	842.1	0.004
Gln	1282.1	1392.4	1747.6	1695.9	2340.5	1937.5	2335.8	0.02
Methionine_sulfoxide	1.4	1.3	1.4	1.8	1.8	2.4	2.0	0.01
Cit		0.0	0.0	0.0	0.0	0.0	0.0	
Orn	0.2	2.3	4.3	2.8	2.9	2.5	2.8	0.36
Pro	238	215.2	239.3	245.2	300.9	287.2	291.3	0.02
2AE								
Lys	262.1	299.6	291.6	325.6	363.6	387.7	352.3	0.02
Lactate								
Uracil								
Citrate	16.7	9.6	27.6	29.1	45.9	35.8	45.4	0.02
Uric_acid								
Opro	93	81.3	81.8	158.3	149.0	215.0	157.1	0.1
Uridine		0.0	0.0	0.0	0.0	0.0	0.0	
Choline	5.9	12.9	11.1	12.5	12.5	14.8	12.0	0.45
Putrescine	0							
Thymidine								
Cytidine								
Deoxycytidine								
Biotin	0.5	0.5	0.6	0.5	0.5	0.5	0.8	0.34
Phe	133.1	143.2	135.5	154.4	168.6	174.6	167.2	0.02
Glucose	4253.9	4054.2	3104.4	2001.2	1370.8	2173.8	1167.4	0.01
Asn	571.8	588.2	525.2	502.8	521.4	534.2	456.1	0.1
Asp	166.6	142.1	97.2	68.9	27.6	61.6	16.9	0.004
Ser	158.2	155.3	130.2	112.5	122.7	134.3	120.6	0.1
Glyceric_acid								
Glu	397.2	418.6	285.1	209.1	103.0	176.3	52.7	0.01
Ascorbate	81.1							
Pyruvate	571.4	458.9	345.0	254.9	140.1	192.3	138.0	0.004
Arg	425.2	417.1	356.1	335.4	306.3	367.8	296.2	0.02
Glutathione	40.9	41.0	34.8	24.1	31.1	26.7	22.2	0.02
Val	307.8	278.5	204.5	150.8	100.8	164.4	90.1	0.01
Niacinamide	6.3	6.4	5.3	5.0	4.8	5.3	3.9	0.05
Oxidized_glutathione	486	483.7	463.0	421.0	416.0	410.2	350.4	0.002
Riboflavin	0.5	0.5	0.4	0.5	0.5	0.4	0.3	0.07
Pyridoxal	8.1	8.5	8.3	4.4	3.9	3.5	2.7	0.01
Ile	373.7	331.0	200.7	147.9	62.3	146.3	54.5	0.004
Leu	379.4	329.8	203.6	149.8	71.3	153.2	72.5	0.02

Supplementary Table 3: Concentrations of medium components adjusted with data of Day 3_wo and Day 7_wo, shown in Supplementary Table 2. P values were obtained by Jonckheere-Terpstra test.

Components	Abbrev.	Day0	Day3	Day7	Day17	Day21	Day24	Day28	P value
Threonine	Thr	100	104.6	96.8	107.4	115.1	115.9	111.7	0.05
Histidine	His	100	105.5	101.9	101.7	119.9	124.5	115.5	0.1
Methionine	Met	100	103.4	95.7	95.5	105.8	108.8	101.8	0.45
Tryptophan	Trp	100	104	95	103.3	108.2	114.2	108.3	0.05
Cystyl-Cysteine	CysCys	100	114.1	119.9	196.1	200.1	235.4	208.7	0.004 *
Glycine	Gly	100	107.8	123.3	134	179.1	161.7	197	0.004 *
Glutamine	Gln	100	108.6	136.3	132.3	182.6	151.1	182.2	0.02 *
Alanine	Ala	100	139.1	136.2	127.9	137.7	150.7	148.8	0.1
Proline	Pro	100	90.4	100.5	103	126.4	120.6	122.4	0.02 *
Lysine	Lys	100	114.3	111.3	124.2	138.8	147.9	134.4	0.02 *
Citrate	Citrate	100	57.5	164.8	174.3	274.1	214.3	271.2	0.02 *
Tyrosine	Tyr	100	103.7	104.5	112.2	121.6	122.5	123.3	0.002 *
Phenylalanine	Phe	100	107.6	101.8	116	126.7	131.2	125.7	0.02 *
Glucose	Glucose	100	95.3	73	47	32.2	51.1	27.4	0.01 $^{\#}$
Asparagine	Asn	100	102.9	91.8	87.9	91.2	93.4	79.8	0.1
Aspartic acid	Asp	100	85.3	58.4	41.4	16.6	37	10.1	0.004 $^{\#}$
Serine	Ser	100	98.2	82.3	71.1	77.6	84.9	76.2	0.1
Glutamic acid	Glu	100	105.4	71.8	52.6	25.9	44.4	13.3	0.01 $^{\#}$
Pyruvate	Pyruvate	100	80.3	60.4	44.6	24.5	33.7	24.1	0.004 $^{\#}$
Arginine	Arg	100	98.1	83.8	78.9	72	86.5	69.7	0.02 $^{\#}$
Valine	Val	100	90.5	66.4	49	32.7	53.4	29.3	0.01 $^{\#}$
Isoleucine	Ile	100	88.6	53.7	39.6	16.7	39.1	14.6	0.004 $^{\#}$
Leucine	Leu	100	86.9	53.6	39.5	18.8	40.4	19.1	0.02 $^{\#}$

Supplementary Table 4: Relative concentration change of medium components, corresponding to the graph of Figure 3a. Statistical analysis was performed with Jonckheere-Terpstra test. P value less than 0.05 was considered significant. Based on this, substances were classified into three groups; produced, stable, and consumed. P values of produced and consumed group were marked with * and $^{\#}$, respectively.

Substances supplemented		Concentration added (µg/mL)
D(+)-Glucose		1000
ITS	Insulin	1
	Transferrin	0.55
	Sodium Selenite	0.00067
L-Aspartic acid (Asp)		30
L-Glutamic Acid (Glu)		75
L-Valine (Val)		46
L-Isoleucine (Ile)		52
L-Leucine (Leu)		52

Supplementary Table 5: Substances augmented in Rat medium 1.

Rat medium 2		
AlbuMAX I	20	mg/mL
Testosterone	1	µM
3,3′,5-Triiodo-L-thyronine sodium	2	ng/mL
LH	1	ng/mL
FSH	1	ng/mL
L-Ascorbic acid 2 glucoside	0.5	mM
DL-α-Tocopherol acetate	0.5	mM
L-Glutathione reduced	0.5	mM
L-α-Lysophosphatidylcholine	100	µg/mL
Lysophosphatidic acid	10	µM
D(+)-Glucose	1	mg/mL
Insulin *	1	µg/mL
Transferrin *	0.55	µg/mL
Sodium Selenite *	0.00067	µg/mL
L-Valine	46	µg/mL
L-Isoleucine	52	µg/mL
L-Leucine	52	µg/mL

Ingredients were added to the α-MEM.
* These 3 were added as ITS (Thermo Fisher Scientific).

Supplementary Table 6: Composition of rat medium 2

Female	Male	Number of Deliveries	Number of Pups per Delivery
Slc:SD	ROSI#1	2	15, 13
Slc:SD	ROSI#2	2	11, 17
ROSI#4	ROSI#3	1	3*

* Note: Loss of pups due to maternal behavior

Supplementary Table 7: Summary of mating result with four ROSI-derived animals.
